# BIRC5 modulates keratinocyte proliferation and apoptosis via PI3K/AKT/mTOR-mediated autophagy

**DOI:** 10.1038/s41598-026-51093-x

**Published:** 2026-04-29

**Authors:** Cuilin Xie, Haixia Wu, Yongjun Chen, Ailing Zou

**Affiliations:** 1 Department of Dermatology, Huangshi Central Hospital, Affiliated Hospital of Hubei Polytechnic University, Huangshi, 43500 Hubei China; 2 Hubei Key Laboratory of Kidney Disease Pathogenesis and Intervention, Huangshi, 435000 Hubei China

**Keywords:** BIRC5, Autophagy, PI3K/AKT/mTOR pathway, HaCaT cells, Cancer, Cell biology, Diseases, Drug discovery, Molecular biology

## Abstract

Psoriasis is characterized by abnormal keratinocyte proliferation and BIRC5, also known as survivin, plays a critical role in the pathogenesis of psoriasis. However, the regulatory role of BIRC5 in psoriasis progression remains unclear. To elucidate the mechanisms by which BIRC5 regulates psoriasis, we used a mouse model of imiquimod (IMQ)-induced psoriasis characterized by psoriasis area and severity index (PASI) scores and epidermal hyperplasia. Immunohistochemistry and western blotting were performed to assess cell proliferation, autophagy, and apoptosis in vivo. The M5 (Oncostatin-M, IL-22, TNF-α, IL-1α, and IL-17 A) was used to treat HaCaT cells for the psoriatic cell model. M5-triggered HaCaT cells were used to investigate the function of BIRC5 in cell proliferation, apoptosis, and autophagy in vitro. The deletion of BIRC5 alleviated IMQ-induced psoriasis-like skin lesions, reduced the PASI scores, and decreased epidermal thickness. Inhibition of BIRC5 suppressed proliferation, facilitated apoptosis, and activated autophagy in these IMQ-induced mice. Consistent with the animal experiment results, M5 accelerated the proliferation of HaCaT cells, whereas inhibited the apoptosis and autophagy of HaCaT cells; however, BIRC5 knockdown partially reversed these effects. Additionally, silencing BIRC5 inhibited the phosphorylation of PI3K, AKT, and mTOR. Furthermore, 3-MA or SC79 treatment negated the autophagy- and apoptosis-enhancing effect of BIRC5 inhibition, as well as the inhibitory effect on HaCaT cell proliferation. Collectively, BIRC5 may accelerate keratinocyte proliferation and inhibit keratinocyte apoptosis through PI3K/AKT/mTOR-mediated autophagy.

**Supplementary Information:**

The online version contains supplementary material available at 10.1038/s41598-026-51093-x.

## Introduction

Psoriasis is an immune-mediated inflammatory skin disease characterized by thickened skin and red patches that can appear anywhere on the body^1,2^. Because psoriasis is recurrent and linked to cardiovascular disease, metabolic syndrome, and other comorbidities, many patients face substantial psychological and economic burdens^3^. The most advanced treatments for psoriasis, including small-molecule targeted therapies and biological agents, have demonstrated promising efficacy compared with that of conventional therapies^4^. However, their high cost and potential systemic side effects restrict their widespread clinical application. Therefore, identifying potential therapeutic targets for psoriasis treatment is imperative.

Autophagy maintains metabolic balance, ensures organelle quality control, and plays a multifaceted role in regulating inflammation and immune responses^5–7^. Dysfunctional autophagy is associated with various disorders^8,9^. Autophagy protects against inflammatory diseases, such as liver injury^10^, inflammatory bowel disease^11^, atherosclerosis^12^, and chronic rhinosinusitis^13^. Cis-khellactone treatment is reported to enhance autophagy and alleviate imiquimod (IMQ)induced psoriasis by inhibiting pro-inflammatory macrophages, thus highlighting the anti-inflammatory effects of autophagy on psoriasis^14^. Further, multiple signaling pathways modulate autophagy. With deepening research, PI3K/AKT/mTOR signaling has gradually become a hotspot for psoriasis research. For instance, tripartite motif-containing 22 (TRIM22) activates PI3K/AKT/mTOR signaling to accelerate psoriasis by promoting cell inflammation and proliferation and suppressing autophagy^15^. Maternally expressed gene 3 (MEG3) inhibits the PI3K/AKT/mTOR axis, accelerating autophagy and restraining inflammation in psoriatic mice^16^. Notably, the PI3K/AKT/mTOR pathway is implicated in key cellular processes including apoptosis, proliferation, and autophagy, which play integral roles in psoriasis progression^17^. However, the specific roles of autophagy and PI3K/AKT/mTOR axis in psoriasis remain to be explored.

BIRC5(also known as survivin), a member of the apoptosis inhibitor protein (IAP) family, was discovered in 1997^18^. BIRC5 expression is enhanced in embryonic tissues and tumors and is linked with tumor cell proliferation, differentiation, and metastasis^19,20^. Interestingly, our previous study revealed upregulated BIRC5 levels in lesional skin samples from patients with psoriasis vulgaris, suggesting that BIRC5 may influence psoriasis development by regulating autophagy^21^. Further, BIRC5 expression is reported to be enhanced in psoriasis skin lesions^22^. However, the role of BIRC5 in psoriasis progression and its regulation through the PI3K/AKT/mTOR pathway remains unclear.

This study aimed to investigate the potential of BIRC5 to aggravate IMQ-induced skin lesions by inhibiting autophagy. Specifically, we elucidated the role of the BIRC5/PI3K/AKT/mTOR axis in autophagy regulation, thereby providing new insights into the therapeutic potential of BIRC5 in IMQ-induced skin lesions.

## Materials and methods

### Ethics approval

All animal experiments were carried out in accordance with the principles of the Guidelines for the Care and Use of Animals and were approved by the Biology and Medicine Ethics Committee of Hubei Polytechnic University (No.2025-2-10 A). The animal research reports adhered to the ARRIVE guidelines.

### Cell culture and treatment

HaCaT cells (SUNNCELL, Wuhan, China) were cultured in DMEM (Bio-Channel, Nanjing, China) with 10% FBS (Bio-Channel) in an environment with 5% CO_2_ at 37 °C. For the psoriatic cell model^23^, HaCaT cells (6 × 10^5^/well) were cultured in 6-well plates for 24 h and treated with 10 ng/mL M5 cocktail solution for another 24 h. The M5 was composed of Oncostatin-M, IL-22, TNF-α, IL-1α, and IL-17 A (MedChemExpress, 10 ng/mL). The control and psoriasis model (M5) groups corresponded to untreated and treated cells, respectively. For cell transfection, negative control shRNA (sh-NC: CCG GGA TTC TCC GAA CGT GTC ACG TCT CGA GAC GTG ACA CGT TCG GAG AAT CTT TTT T) and BIRC5 shRNA (sh-BIRC5: CCG GTA GGA AAG GAG ATC AAC ATT TCT CGA GAA ATG TTG ATC TCC TTT CCT ATT TTT T) were synthesized by Transheep (Shanghai, China). Transfection was performed following the completion of the cell modeling experiment. HaCaT cells were transfected with sh-BIRC5 or sh-NC using Lipofectamine 2000 for 48 h. The experimental groups are as follows: Control group, M5 group, M5 + sh-NC group, and M5 + sh-BIRC5 group. To explore the mechanism PI3K/AKT/mTOR and autophagy in vitro, M5-stimulated HaCaT cells were exposed to 3-MA at 5 mM (MedChemExpress) for 4 h or SC79 at 10 µM (MedChemExpress) for 1 h, cor-respectively.

### Cell proliferation assay

The cell proliferation assay was conducted based on the predefined experimental groups. The detailed pro-cedures are outlined below.HaCaT cells were treated with 100 µM of BrdU (ST1056, Beyotime) for one hour. After fixation with 4% polyformaldehyde for 15 min, the cells were blocked with 5% bovine serum albumin and incubated with an anti-BrdU antibody for 30 min. After nuclear staining for 5 min, BrdU-positive cells were imaged using a fluorescence microscope (OLYMPUS, Japan). The experimental groups are as follows: Control group, M5 group, M5 + sh-NC group, and M5 + sh-BIRC5 group, M5 + sh-BIRC5 + 3-MA group, and M5 + 3-MA group. The cells in each experimental group were pro-cessed sequentially following the aforementioned steps.

### Flow cytometry analysis (Annexin V/PI Apoptosis Assay)

HaCaT cell apoptosis was assessed using an Annexin V-FITC/Propidium Iodide (PI) kit (C1062L, Beyotime). The HaCat cells were digested with trypsin and their density was adjusted to 5 × 10^4^. Then collect 50,000–100,000 resuspended cells and spin down at 1000 × g for 5 min. Aspirate the supernatant thoroughly. Reconstitute the pellet in 195 µL Annexin V-FITC binding buffer with gentle pipetting. Supplement sequentially with 5 µL of Annexin V-FITC and 10 µL of PI at 25 °C for 20 min, followed by immediate transfer to a pre-chilled ice bath to arrest further staining, and test immediately on the machine (Annexin V-FITC, green fluorescence and PI, red fluorescence). Apoptosis was detected using flow cytometry (Dxp Athena, Cytek) and analyzed using FlowJo software (BD Biosciences, USA).

### Epidermis separation for protein extraction

To enrich epidermal proteins for western blot analysis, mouse skin tissues were processed using a heat-separation method. Briefly, freshly excised dorsal skin samples were rinsed with cold phosphate-buffered saline (PBS) and incubated in PBS at 56 °C for 2–5 min. After incubation, the epidermis was gently separated from the dermis using sterile forceps. The isolated epidermal tissues were immediately collected and lysed in RIPA buffer containing PMSF for protein extraction. This method enables rapid enrichment of epidermal tissue while minimizing protein degradation associated with prolonged enzymatic digestion. The collected samples therefore predominantly represent epidermal proteins, although minor dermal contamination cannot be completely excluded.

### Western blotting (WB)

HaCaT cells and epidermis-enriched skin tissues were lysed using RIPA buffer with 1% PMSF (Beyotime, Shanghai, China) to obtain total protein. The lysates were separated using 10% sodium dodecyl sulfate-polyacrylamide gel electrophoresis and transferred onto PVDF membranes. Next, the membranes were sealed with 5% non-fat milk, incubated with primary antibodies, and labeled with secondary antibodies. Immunoblots were visualized using enhanced chemiluminescence solution (Beyotime, Shanghai, China). The antibodies used are listed in Table 1.

**Table 1:** Primary antibodies used in western blotting assay.

Antibody	Company (Cat. No.)	Working dilution
PCNA	Proteintech (10205-2-AP)	1:5000
BIRC5	Proteintech (10508-1-AP)	1:1000
Bax	Abcam (ab32503)	1:2000
cleaved Caspase 3	Abcam (ab2302/ab214430)	1:2000/1:5000
Bcl-2	Proteintech (12789-1-AP/68103-1-Ig)	1:2000/1:5000
β-actin	Proteintech (20536-1-AP)	1:5000
LC3B	Proteintech (81004-1-RR)	1:5000
p62	Proteintech (18420-1-AP)/ Abcam (ab314504)	1:5000/ 1:1000
p-PI3K	Thermo Fisher Scientific (PA5-104853)	1:2000
PI3K	Cell Signaling Technology (#4292S)	1:1000
p-AKT	Proteintech (66444-1-Ig)	1:2000
AKT	Proteintech (60203-2-Ig)	1:5000
p-mTOR	Proteintech (67778-1-Ig)	1:2000
mTOR	Proteintech (66888-1-Ig)	1:5000
Goat Anti-Rabbit	Proteintech (RGAR001)	1:5000
Goat Anti-Mouse	Proteintech (RGAM001)	1:5000

### Transmission electron microscopy (TEM) analysis

HaCaT cells were fixed in 1% osmium acid for two hours and then washed thrice with 0.1 M phosphoric acid rinse solution (15 min per wash). Gradient dehydration was performed with ethanol and/or acetone. The cells were then embedded, ultrathin-sectioned (70 nm), and double-stained with 2% uranyl acetate and lead citrate. Autophagy ultrastructure was observed and photographed using TEM (HT7800; Hitachi, Tokyo, Japan).

### IMQ-induced mouse model of psoriasis

The 6-8-week-old female BALB/c mice (Hunan Slac Jingda Laboratory Animal Co. Ltd.), weighing 18–22 g, were purchased for the animal experiment. The mice were randomly allocated into four groups (*n* = 6/group): Control, IMQ, IMQ + AAV-sh-NC, and IMQ + AAV-sh-BIRC5. These mice were maintained in a temperature-controlled (25 ± 2 °C) and humidi-ty-controlled (50–60%) environment with a 12/12-hour light/dark cycle. Food and water were provided ad libitum during the study. A 7-day acclimation period was implemented before initiating experiments. For IMQ-induced psoriasis mouse model, the animals were topically treated with 62.5 mg of 5% Imiquimod (IMQ) cream (Sichuan Mingxin Pharmaceutical Co., LTD, China, H20030129), daily for 7 consecutive days on shaved 2 cm×3 cm back skin. Adeno-associated viruses (AAV2/9-K14) expressing either sh-BIRC5 (GAT CAC CGA GAA CGA GCC TGA TTT GCT CGA GCA AAT CAG GCT CGT TCT CGG TTT TTT T) or sh-NC (GAT CGA TTC TCC GAA CGT GTC ACG TCT CGA GAC GTG ACA CGT TCG GAG AAT CTT TTT T) were obtained from HANBio (Shanghai, China). The shRNA expression cassettes were driven by the keratinocyte-specific K14 promoter to achieve preferential gene silencing in epidermal keratinocytes. A GFP reporter was incorporated into both vectors (AAV-K14-GFP-U6-sh-BIRC5 and AAV-K14-GFP-U6-sh-NC) to visualize viral transduction efficiency in skin tissues. For BIRC5 inhibition, five points on the central back skin of mice were injected with 20 µL of AAV-sh-NC or sh-BIRC5 (1 × 10^12^ vg/mL) per point via the intradermal injection. The injection points were spaced approximately 0.5 cm from one another, with the injection carried out at a depth ranging from 1 to 2 mm. And the injection was given two weeks before IMQ-induced psoriasis mouse model. After 7 days of continuous topical application of IMQ, mice were euthanized through CO_2_ inhalation (30–70% vol/min, 5 min) followed by cervical dislocation, and death was verified by cessation of heartbeat and respiration. Following viral injection, the distribution of the GFP signal can be visualized by the green fluorescence under a fluorescence microscope. All animal experiments were approved by the Biology and Ethics Committee, Hubei Polytechnic University.

### Psoriasis area and severity index (PASI) evaluation

PASI evaluation was performed to assess the severity of skin lesions for IMQ-induced psoriasis mouse model. Three clinical signs, i.e., scaling, erythema, and thickness, were scored on a scale of 0 to 4 (0, no skin abnormalities; 1, mild; 2, moderate; 3, severe; 4, very severe). The cumulative score, ranging from 0 to 12, indicated the severity of skin lesions and was calculated by summing the scores for scaling, erythema, and thickness.

### Histological assessment

The mouse skin samples were fixed in formalin and embedded in paraffin. Subsequently, the tissue samples were sliced into 4 μm sections, placed on slides, and stained with hematoxylin and eosin (H&E). In parallel, frozen sections were prepared and examined for viral fluorescence using a Nikon inverted fluo-rescence microscope. Epidermal thickness was measured at five random locations on HE-stained images using ImageJ software and averaged.For immunohistochemistry, the skin sections were stained for BIRC5 (ab134170, 1:800, Abcam) and Ki-67 (28074-1-AP, 1:1000; Proteintech, Wuhan, China). Images were captured using a microscope for further analysis.

### Statistical analysis

Data analysis was conducted using GraphPad Prism 8.0 (La Jolla, CA, USA) software, with measurement data expressed as the mean ± standard deviation. All experiments were performed in triplicate. An unpaired T-test was used to compare differences between two independent samples with normal distribution; non-parametric test was employed for comparisons between two independent samples with non-normal distribution. For comparisons among more than two groups, we employed one-way analysis of variance (ANOVA). When the ANOVA indicated significant differences, we performed post hoc pairwise comparisons using Tukey’s honestly significant difference (HSD) test. Statistical significance was set at *P* < 0.05 (^*^P < 0.05; ^**^P < 0.01; ^***^P < 0.001).

## Results

### BIRC5 knockdown alleviated psoriasis-like skin lesions

**Fig. 1:**
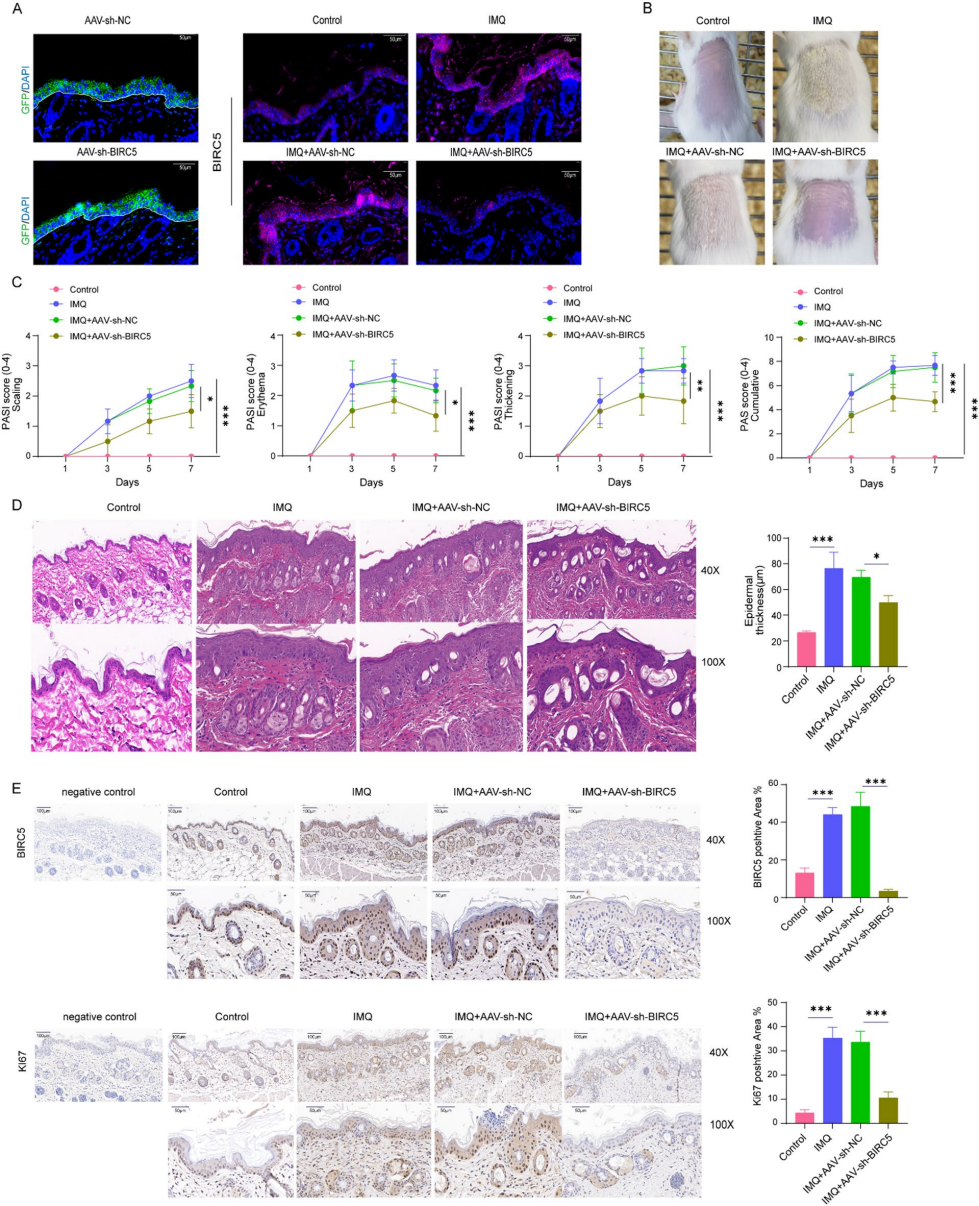

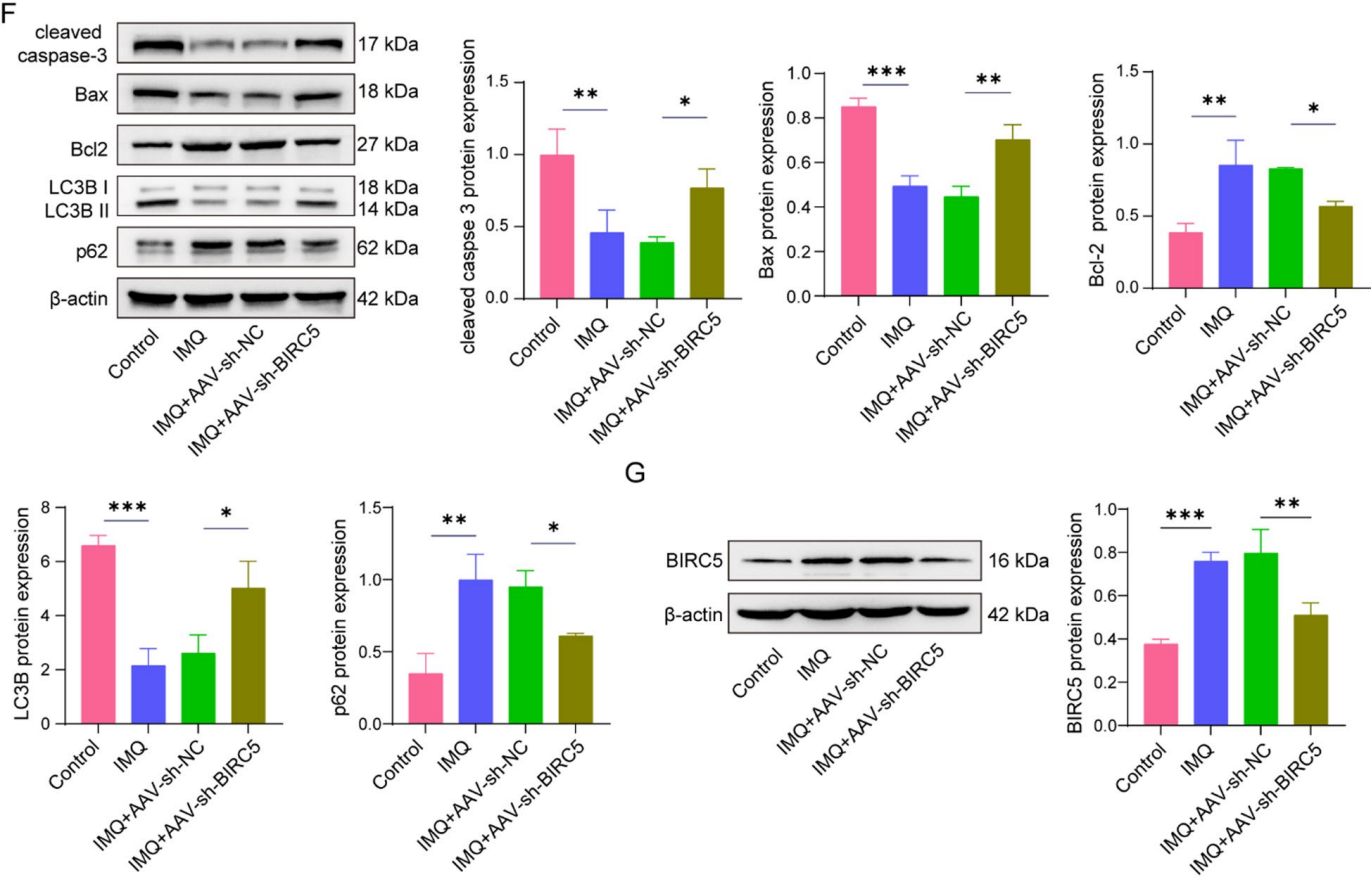
Inhibition of BIRC5 alleviated psoriasis-like skin lesions. This experiment was divided into the following groups: Control, IMQ, IMQ + AAV-sh-NC, and IMQ + AAV-sh-BIRC5. (**A**) Fluorescent images showing AAV transduction efficiency and immunofluorescence staining of BIRC5 in mouse dorsal skin. (**B**) Images of the mouse dorsal skin on days 7 post-IMQ treatment alone or together with AAV-sh-BIRC5. (**C**) The assessment of the psoriasis area and severity index (PASI) scores in different groups. (**D**) Hematoxylin and eosin (H&E) staining of the mouse dorsal skin sections in different groups. (**E**) BIRC5 and Ki-67 expression were measured by immunohistochemistry assay in different groups. (**F**) Cleaved Caspase 3, Bax, Bcl-2, LC3B, and p62 protein levels were measured by WB in different groups. (G) BIRC5 protein levels were measured by WB in different groups. *n* = 6. ^*^P < 0.05, ^**^P < 0.01, and ^***^ *P* < 0.001.

To assess the effects of BIRC5 on psoriatic skin lesions, an IMQ-induced psoriasis-like mouse model was generated and injected with or without adeno-associated virus (AAV)-sh-BIRC5. As shown in fluorescent images in Fig. 1A, successful transduction and expression of AAV-sh-BIRC5 in the dorsal skin of mice were confirmed by GFP green fluorescence signals; Furthermore, IMQ induced upregulation of BIRC5 in epidermal tissues, which was significantly inhibited by AAV-sh-BIRC5. As depicted in Fig. 1B, after seven days’ IMQ administration, the mice showed symptoms of erythema, desquamation, and increased epidermal thickness. Skin lesions in mice were significantly reduced after BIRC5 silencing (Fig. 1B). Additionally, psoriasis area and severity index (PASI) scores (scaling, erythema, thickening, and cumulative) distinctly increased in the IMQ group and the PASI scores were significantly reduced after BIRC5 knockdown in the IMQ + AAV-sh-BIRC5 group (Fig. 1C). Furthermore, H&E staining revealed that the epidermal skin layer of mice in the IMQ group was thickened and showed hyperkeratosis. These symptoms were significantly reduced after BIRC5 knockdown in the IMQ + AAV-sh-BIRC5 group (Fig. 1D). Figure 1E showed that BIRC5 immunoreactivity was markedly increased in IMQ-induced mice, with strong staining observed throughout the epidermis, particularly in basal keratinocytes. In contrast, BIRC5 expression in normal skin was detectable but relatively weak and mainly confined to the epidermal basal layer. Notably, both BIRC5 and Ki-67 levels were enhanced in IMQ-induced mice, and these were alleviated by BIRC5 knockdown in the IMQ + AAV-sh-BIRC5 group, which confirmed that knockdown of BIRC5 was effective in IMQ-induced mouse model (Fig. 1E). As indicated in Fig. 1G, sh-BIRC5 were successfully knocked down in the mouse model. When compared with the control group in WB experiment, the IMQ + AAV-sh-BIRC5 group exhibited elevated expression levels of cleaved caspase 3, Bax, and LC3B. Conversely, the expression levels of Bcl-2 and p62 (Fig. 1F) were downregulated in the IMQ + AAV-sh-BIRC5 group. These changes were accompanied by statistically significant differences (Fig. 1F). Figure 1F demonstrated that BIRC5 knockdown promoted apoptosis and activated autophagy. These above findings suggest that BIRC5 knockdown suppressed proliferation, facilitated apoptosis, and initiated autophagy, consequently ameliorating psoriasis-like skin lesions.

### Silencing of BIRC5 inhibited proliferation and facilitated apoptosis in M5-triggered HaCaT cells

**Fig. 2:**
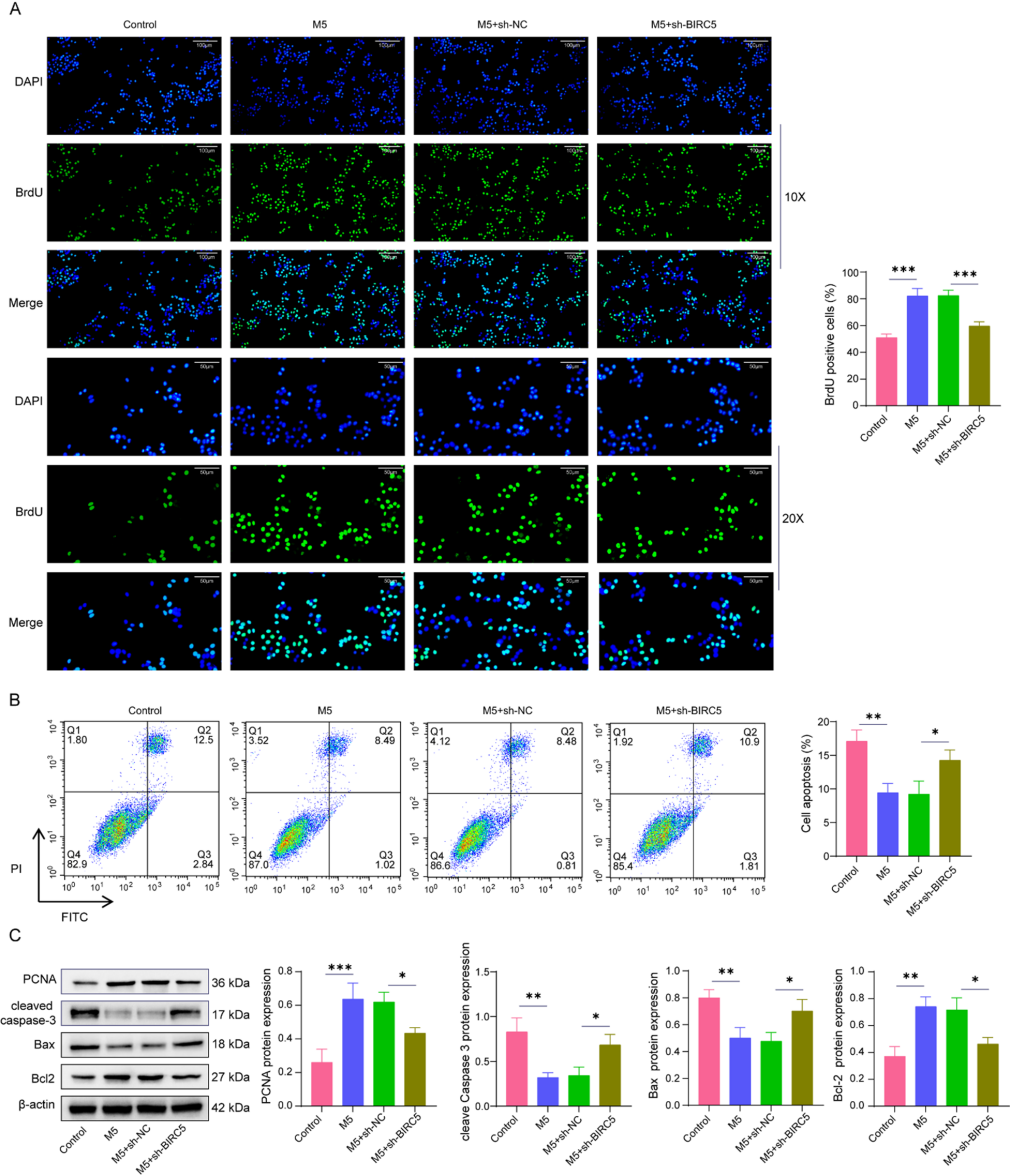
Silencing of BIRC5 inhibited proliferation and facilitated apoptosis in M5-triggered HaCaT cells. The HaCaT cells were divided into the following groups: Control, M5, M5 + sh-NC, and M5 + sh-BIRC5. (**A**) BrdU assay was used to detect cell proliferation in the above groups. (**B**) Flow cytometry was used to detect cell apoptosis in the above groups. (**C**) PCNA, cleaved Caspase 3, Bax, and Bcl-2 protein levels were detected by WB. *n* = 3. ^*^P < 0.05, ^**^P < 0.01, and ^***^ *P* < 0.001.

As illustrated in Fig. 2A, the proportion of BrdU positive cells in the M5 **+** sh-BIRC5 group was notably lower than that in the control group. BIRC5 inhibition suppressed M5-induced proliferation of HaCaT cells. Whereas, the results of Annexin V/PI Apoptosis Assay showed that the fraction of apoptosis cells in the M5 + sh-BIRC5 group was significantly higher than that in the control group (Fig. 2B). Proliferating cell nuclear antigen (PCNA) is involved in cellular DNA synthesis and primarily used as a proliferation status indicator^24^. PCNA and Bcl-2 levels were enhanced while cleaved Caspase 3 and Bax levels were decreased in M5-triggered HaCaT cells, which were counteracted in the M5 + sh-BIRC5 group (Fig. 2C). In summary, Silencing of BIRC5 suppressed proliferation and facilitated apoptosis in M5-triggered HaCaT cells.

### Silencing of BIRC5 initiated autophagy in M5-stimulated HaCaT cells

**Fig. 3:**
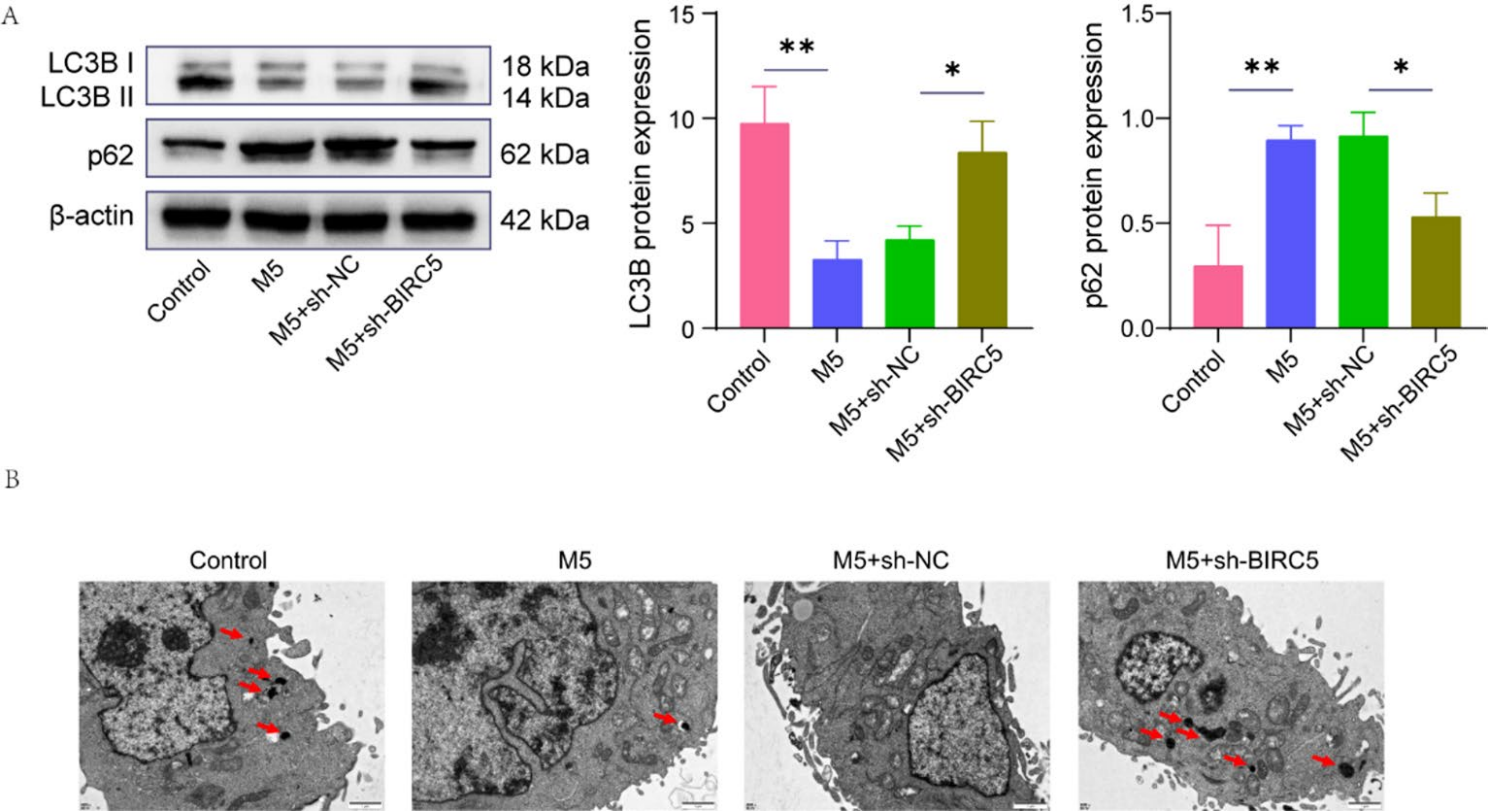
Knockdown of BIRC5 facilitated autophagy in M5-stimulated HaCaT cells. The HaCaT cells were divided into the following groups: Control, M5, M5 + sh-NC, and M5 + sh-BIRC5. (**A**) LC3B and p62 pro-tein levels were detected by WB in different groups. (**B**) TEM was performed to observe autophagosome formation in the above groups. *n* = 3. ^*^P < 0.05, ^**^P < 0.01, and ^***^ *P* < 0.001.

As opposed to the control group, the M5 + sh-BIRC5 group showed an increase in LC3B expression and a decrease in p62 expression (Fig. 3A). Moreover, the TEM results indicated that the autophagosome formation was reduced in M5-stimulated HaCaT cells, whereas the autophagosome formation was increased in the M5 + sh-BIRC5 group (Fig. 3B). These results demonstrated that silencing of BIRC5 initiated autophagy in M5-stimulated HaCaT cells.

### BIRC5 knockdown suppressed proliferation and facilitated apoptosis by activating autophagy

**Fig. 4:**
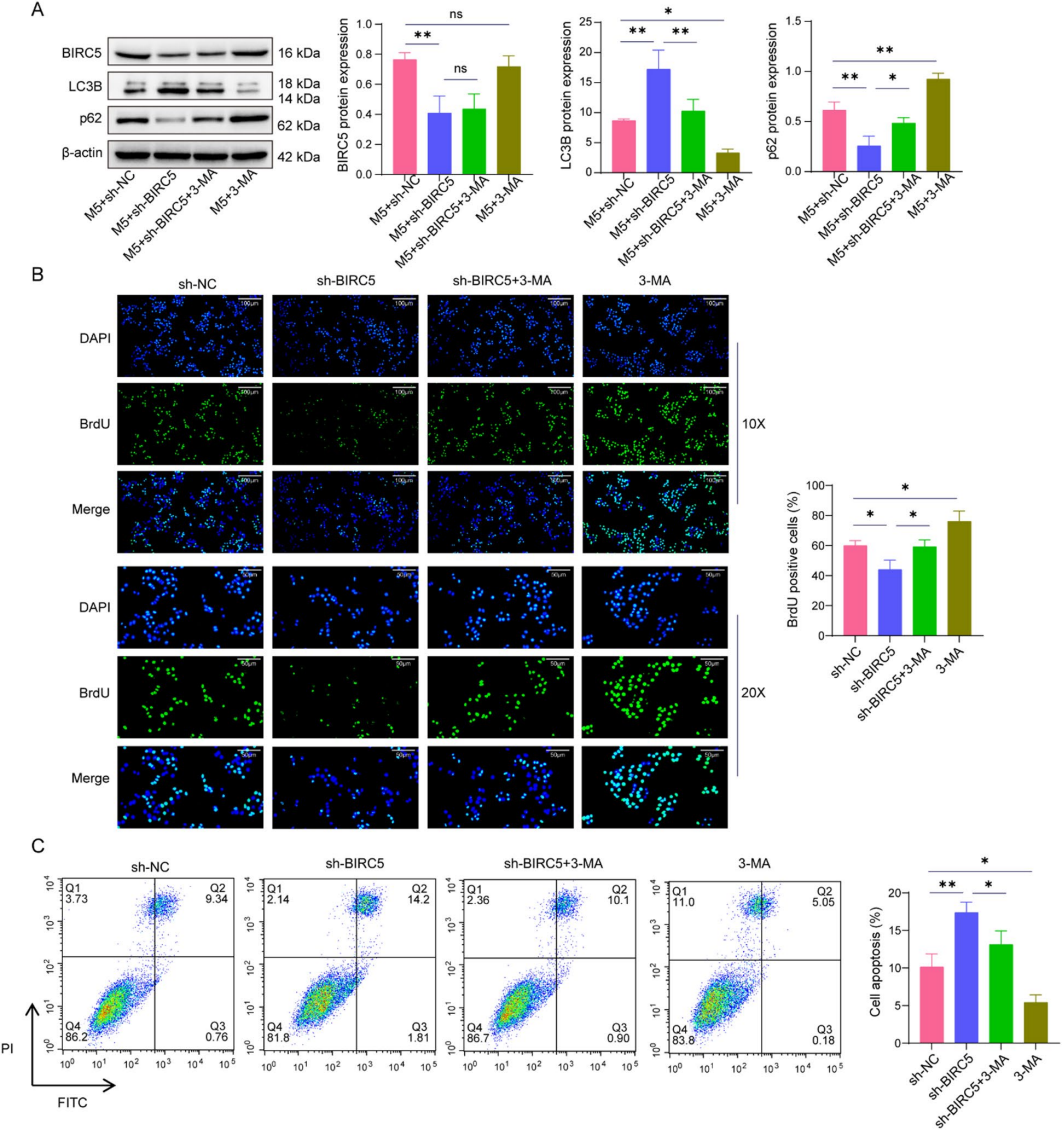
(**A**) BIRC5, LC3B, and p62 protein levels were detected by WB in different groups. (**B**) BrdU assay was used to detect cell proliferation in the above groups. (C) Flow cytometry was used to detect cell apoptosis in the above groups. *n* = 3. ^*^P < 0.05, ^**^P < 0.01, and ^***^ *P* < 0.001.

An autophagy inhibitor (3-MA) was used to treat BIRC5-silenced M5-triggered HaCaT cells. The M5- triggered cells were divided into the following groups: sh-NC, sh-BIRC5, sh-BIRC5 + 3-MA, 3-MA. The WB results showed that BIRC5 protein levels were significantly decreased in the M5 + sh-BIRC5 group, compared with the M5-stimulated group, which validated that knockdown of BIRC5 was effective in M5-stimulated HaCaT cells (Fig. 4A). As depicted in Fig. 4A, in contrast to the M5 + sh-BIRC5 group, p62 protein levels were reduced, whereas LC3B levels were enhanced in the M5 + sh-BIRC5 + 3-MA group, and these were neutralized by 3-MA. The BrdU results displayed that the proportion of positive cells was higher in the M5 + sh-BIRC5 + 3-MA group than that in the M5 + sh-BIRC5 group, which was partially reversed by 3-MA (Fig. 4B). Meanwhile, the fraction of apoptosis cells was lower in the M5 + sh-BIRC5 + 3-MA group than that in the M5 + sh-BIRC5 group (Fig. 4C). These results revealed that BIRC5 knockdown suppressed proliferation and facilitated apoptosis by activating autophagy in M5-induced HaCaT cells.

### BIRC5 knockdown inactivated PI3K/AKT/mTOR pathway

**Fig. 5:**
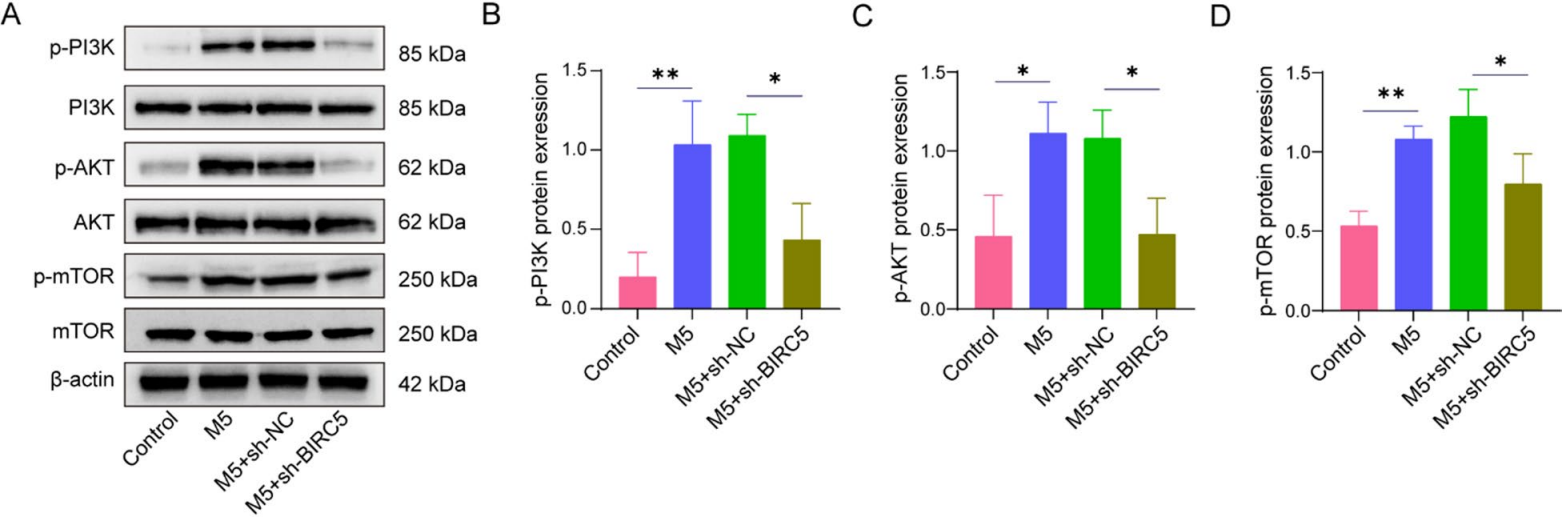
BIRC5 knockdown inactivating PI3K/AKT/mTOR pathway. The HaCaT cells were divided into the following groups: Control, M5, M5 + sh-NC, and M5 + sh-BIRC5. (**A**) p-PI3K, PI3K, p-AKT, AKT, p-mTOR, and mTOR protein levels were detected by WB in different groups. (**B**) The quantitative analysis results of p-PI3K protein levels in different groups. (**C**) The quantitative analysis results of p-AKT protein levels in different groups. (**D**) The quantitative analysis results of mTOR protein levels in different groups. *n* = 3. ^*^P < 0.05, ^**^P < 0.01, and ^***^ *P* < 0.001.

As exhibited in Fig. 5, compared with the control group, the levels of phosphorylated PI3K, AKT, and mTOR were delined in the M5 + sh-BIRC5 group, and the differences were statistically significant after analysis. These data suggested that BIRC5 knockdown inhibited the PI3K/AKT/mTOR axis in M5-triggered HaCaT cells.

### BIRC5 inhibition relieved proliferation and promoted apoptosis by activating autophagy through the PI3K/AKT/mTOR axis

**Fig. 6:**
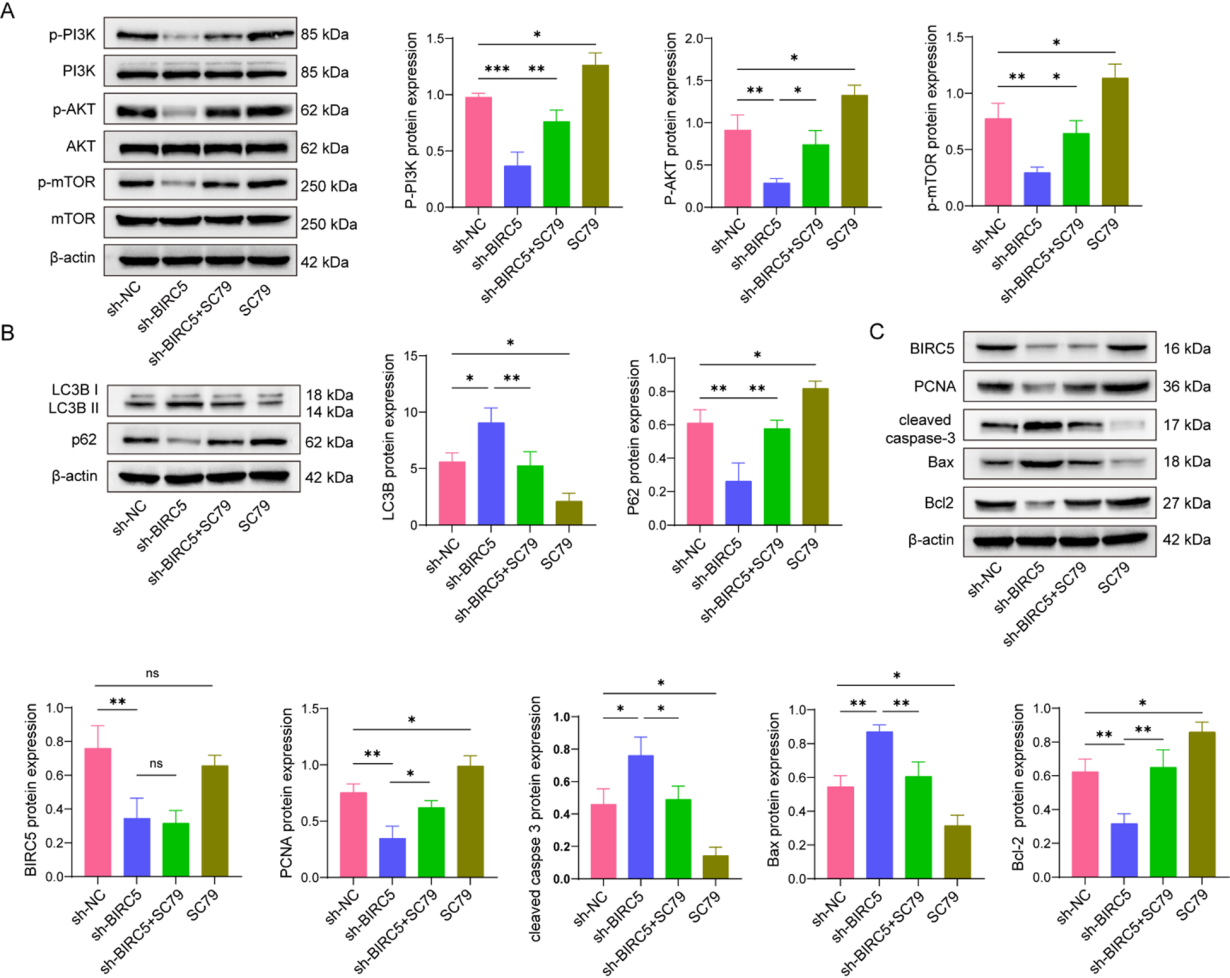
BIRC5 inhibition relieved proliferation and promoted apoptosis by activating autophagy through the PI3K/AKT/mTOR axis. The M5-stimulated cells were divided into the following groups: sh-NC, sh-BIRC5, sh-BIRC5 + SC79, SC79. (**A**) p-PI3K, PI3K, p-AKT, AKT, p-mTOR, and mTOR protein levels were de-tected by WB in different groups. (**B**) LC3B and p62 protein levels were detected by WB in different groups. (**C**) BIRC5, PCNA, cleaved Caspase 3, Bax, and Bcl-2 protein levels were detected by WB in dif-ferent groups. *n* = 3. ^*^P *<* 0.05, ^**^P < 0.01, and ^***^ *P* < 0.001.

To confirm whether BIRC5 inhibition relieved M5-indcuced HaCaT cell proliferation via the PI3K/AKT/mTOR axis, SC79, a PI3K/AKT signaling activator, was applied to treat BIRC5-silenced M5-stimulated HaCaT cells. The M5-stimulated cells were divided into the following groups: sh-NC, sh-BIRC5, sh-BIRC5 + SC79, SC79. BIRC5 silencing decreased the levels of p-PI3K, p-AKT, and p-mTOR in M5-treated HaCaT cells, and these effects were abolished by SC79 treatment (Fig. 6A). Moreover, SC79 partially reversed the upregulation of LC3B and downregulation of p62 induced by BIRC5 silencing in M5-stimulated HaCaT cells (Fig. 6B). Furthermore, BIRC5 silencing enhanced the levels of cleaved caspase 3 and Bax, and decreased the levels of BIRC5, PCNA, and Bcl-2 in M5-stimulated HaCaT cells; these effects were neutralized by SC79 treatment (Fig. 6C). These results indicated that BIRC5 inhibition relieved proliferation and promoted apoptosis by activating autophagy via the PI3K/AKT/mTOR pathway.

## Discussion

Psoriasis arises from a combination of genetic predisposition and environmental triggers such as infections, stress, alcohol, tobacco use, obesity, and certain medications^25^. Excessive proliferation and a lack of apoptosis in keratinocytes are important characteristics of psoriasis. In psoriasis, keratinocyte proliferation is accelerated, thereby thickening the epidermal layer. Meanwhile, apoptosis of keratinocytes decreases, and the cells that should have died and been metabolized normally do not undergo apoptosis as programmed, resulting in typical psoriasis symptoms such as erythema and scales on the skin^26^. BIRC5, also known as survivin, is a key factor involved in keratinocyte apoptosis during psoriasis. Most studies have shown BIRC5 expression is upregulated in psoriatic skin^21,22,27,28^. It is a member of the apoptosis-regulating protein family and inhibits the activity of effector caspases, Caspase-3 and Caspase-7, thereby blocking keratinocyte apoptosis^29^. Furthermore, Bai W et al. propose that BIRC5 could serve as potential therapeutic targets for the prevention and treatment of psoriasis. However, Gunduz K et al. suggest that survivin does not significantly contribute to the epidermal proliferation and thickness observed in psoriasis^30^. The function of BIRC5 in regulating the advancement of psoriasis remains debated. Our study is to investigate the role of BIRC5 in psoriasis pathogenesis. In our study, we confirmed through animal experiments that BIRC5 expression is significantly upregulated in IMQ-induced psoriasis-like skin compared with normal skin, and BIRC5 silencing alleviated psoriasis-related symptoms.

BIRC5 is an IAP that plays a key role in modulating cell division and inhibiting programmed cell death^31^. Its relationship with autophagy, a process of cellular degradation and recycling, is complex and context-dependent^32^. BIRC5 suppresses autophagy under certain conditions^33^. As BIRC5 is primarily anti-apoptotic, it may also inhibit autophagy to promote cell survival, particularly in cancer cells^34^. This allows cancer cells to escape autophagic cell death and continue proliferating^35^. In psoriatic skin lesions, impaired autophagy can lead to excessive inflammasome activation, resulting in the release of large amounts of inflammatory cytokines such as interleukin-1β (IL-1β) and interleukin-18 (IL-18)^36^. These cytokines recruit and activate immune cells, exacerbating the inflammatory response in the skin^36^. Lee et al. reported that autophagy impairment in myeloid cells, particularly macrophages, coupled with IL-1β dysregulation, plays a pivotal role in neutrophilic inflammation and psoriasis development^37^. More importantly, we verified that BIRC5 silencing inhibited proliferation and promoted apoptosis by modulating autophagy in vitro.

In psoriasis, PI3K activation triggers the phosphorylation of a 3-hydroxyl group, subsequently activating AKT kinase through the phosphorylation of Thr308 and Ser473^38^. This process promotes keratinocyte hyperproliferation and inhibits keratinocyte differentiation. AKT is highly activated in the epidermal layers of psoriatic lesions, and promotes cell proliferation in the epidermis^39^. AKT phosphorylation activates several proteins, including those involved in the mTOR signaling pathway, which is tightly regulated by a feedback loop. Previous studies have shown that the inhibition of PI3K/AKT/mTOR signaling can help slow psoriasis progression^40,41^. Consistent with previous findings, we found that the PI3K/AKT/mTOR pathway was activated in M5-triggered HaCaT cells. BIRC5 has been shown to interact with the PI3K/AKT/mTOR axis, a crucial signaling cascade that promotes cell survival, growth, and metabolism^42,43^. This pathway inhibits apoptosis and regulates autophagy, making it essential for cell survival, particularly in cancer cells^44^. AKT activation leads to the inhibition of pro-apoptotic factors and increases the levels of anti-apoptotic proteins, including BIRC5^45^. Consequently, the anti-apoptotic role of BIRC5 can be further enhanced through activation of this pathway, contributing to cancer cell survival^46^. Further, BIRC5 expression promotes tumor angiogenesis via PI3K/AKT signaling^47^. BIRC5 may affect multi-drug resistance in cancer cells via the PI3K/AKT/mTOR axis^48^. As described in our study, BIRC5 knockdown promoted autophagy through the PI3K/AKT/mTOR axis in M5-induced HaCaT cells.

Autophagy, the cellular mechanism of self-degradation and recycling of damaged organelles and proteins, plays a crucial role in the pathogenesis of psoriasis^49^. In psoriasis, dysregulated autophagy contributes to disease development by disrupting keratinocyte homeostasis and exacerbating immune responses^50^. Specifically, impaired autophagy in skin cells leads to the accumulation of cellular debris, which triggers inflammatory cascades involving key cytokines such as TNF-α, IL-17, and IL-23, driving abnormal proliferation of keratinocytes and immune cell infiltration^50^. In our study, our results showed that BIRC5 regulated autophagy through the PI3K/AKT/mTOR pathway, exerting a pathogenic role in psoriasis. Interestingly, 3-MA treatment altered the protein levels of LC3B and p62 but did not affect the level of BIRC5 in Fig. 4A. It maybe that while 3-MA is known to primarily affect autophagy markers (LC3B and p62), BIRC5 (survivin) is an upstream molecule that regulates autophagy through the PI3K/AKT/mTOR pathway. This suggests that the observed changes in LC3B and p62 occur through autophagy-specific mechanisms, while BIRC5 levels are maintained through other regulatory processes.

Therefore, we have now more distinctly emphasized the novel aspects of our findings, including: (1) the evidence of BIRC5 upregulation specifically in the IMQ-induced psoriasis-like mouse model, (2) a thorough examination of its functional impacts in psoriatic keratinocytes, and (3) comprehensively elucidating the role of proliferation, apoptosis, and autophagy regulation in the pathogenesis of psoriasis. In conclusion, our results suggest that BIRC5 may accelerate keratinocyte proliferation and inhibit apoptosis in psoriasis via PI3K/AKT/mTOR-mediated autophagy. In light of the significant contribution of BIRC5 to psoriasis mechanisms, it could be a viable target for therapy, with the understanding that its therapeutic benefits need to be validated in future studies.

Guilloteau K et al. first clearly defined the M5 cytokine cocktail (IL-1α, IL-17 A, IL-22, OSM, TNF-α) was a well-established and valuable in vitro tool for modeling key inflammatory aspects of psoriasis^51^. The M5 model is exceptionally powerful for studying the autonomous inflammatory response of keratinocytes to a defined set of key psoriatic cytokines. Its limitation is that it represents a specific, albeit crucial, snapshot of the disease—the amplified effector phase within keratinocytes—rather than the complete, multi-cellular pathophysiology. Our findings using this model should therefore be interpreted as elucidating mechanisms within this specific inflammatory module, with the understanding that in vivo validation in more complex systems remains essential. Thus, we also performed validation in in vivo models to enhance the credibility of the results.

## Conclusions

This study revealed that BIRC5 silencing restrained proliferation of M5-triggered HaCaT cells and improved skin lesions in IMQ-induced mice. Furthermore, the suppression of cell proliferation, as well as the enhancement of apoptosis and autophagy triggered by BIRC5 knockdown, were significantly associated with the PI3K/AKT/mTOR signaling pathway. These findings reveal that BIRC5 may accelerate keratinocyte proliferation and inhibit keratinocyte apoptosis through PI3K/AKT/mTOR-mediated autophagy.

## Supplementary Information

Below is the link to the electronic supplementary material.

Supplementary Material 1

## Data Availability

The data that support the findings of this study are available from the corresponding authors author upon reasonable request.
