# Supplementary material for: BIRC5 modulates keratinocyte proliferation and apoptosis via PI3K/AKT/mTOR-mediated autophagy
Source: Sci Rep. 2026 Apr 29;16:20062. doi: 10.1038/s41598-026-51093-x (PMC13324518; doi:10.1038/s41598-026-51093-x)
Supplement: Supplementary file 1 — Supplementary Material 1 [file 41598_2026_51093_MOESM1_ESM.pdf]

The original versions of gels/blots in Figure1-6

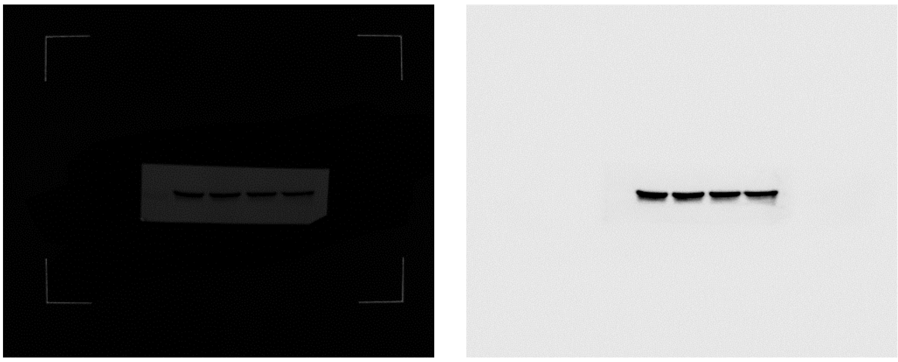

Fig S1 (F) β -actin

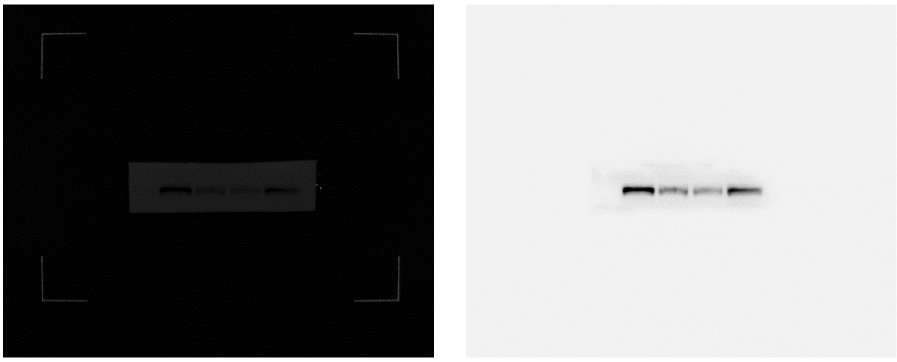

Fig S1(F) Cleaved caspase3

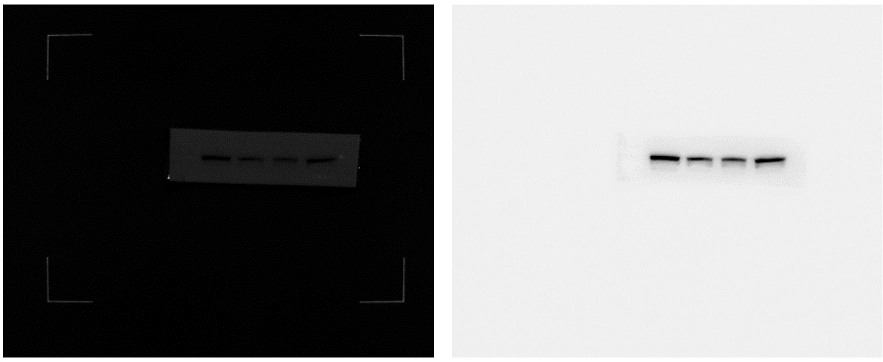

Fig S1(F) Bax

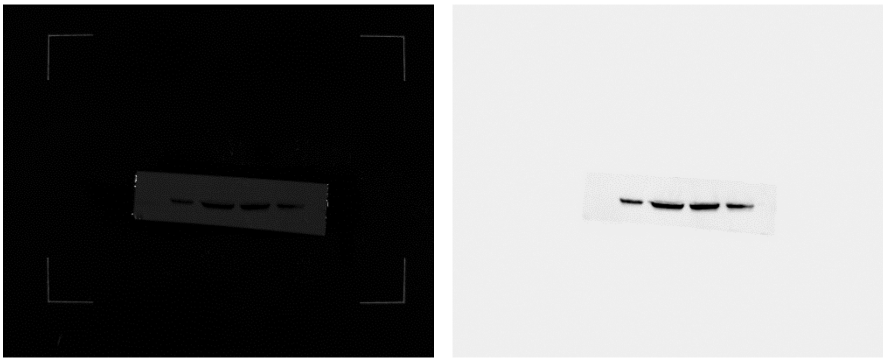

Fig S1(F) Bcl2

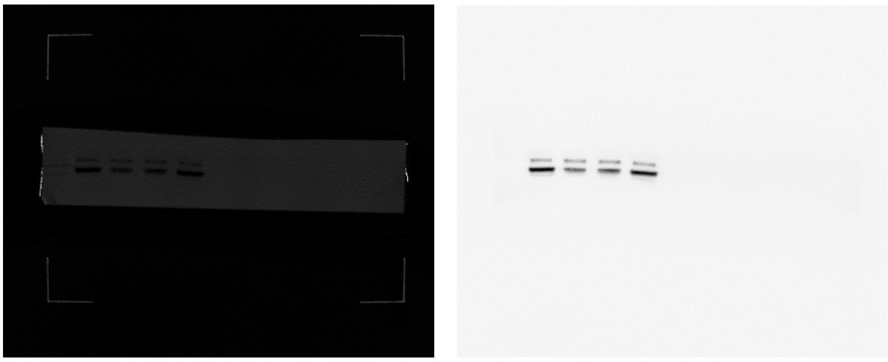

Fig S1(F) LC3BI/LC3BII

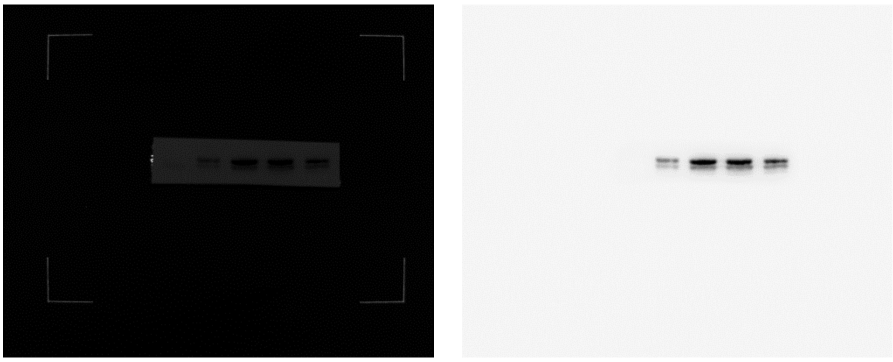

Fig S1(F) p62

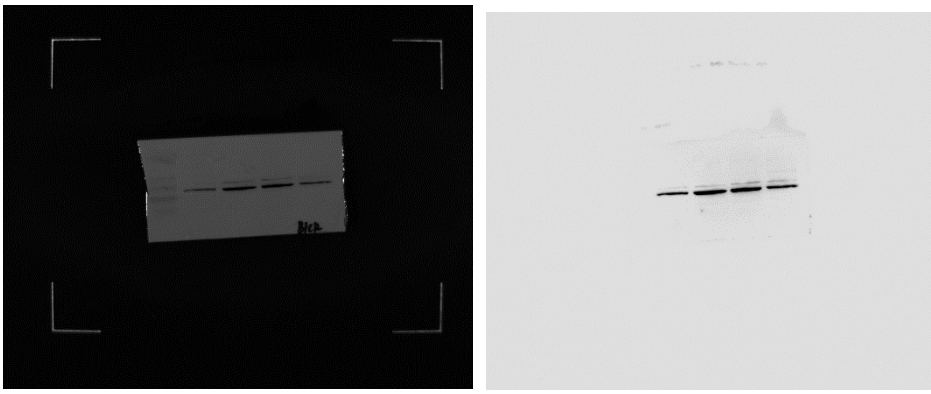

Fig S1(G) BIRC5

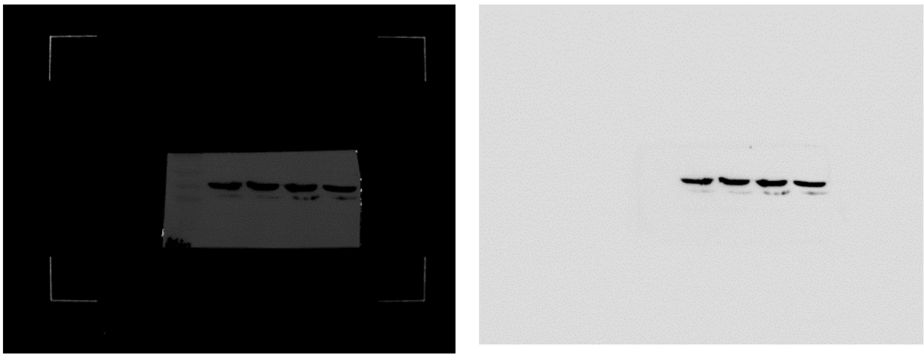

Fig S1(G) β -actin

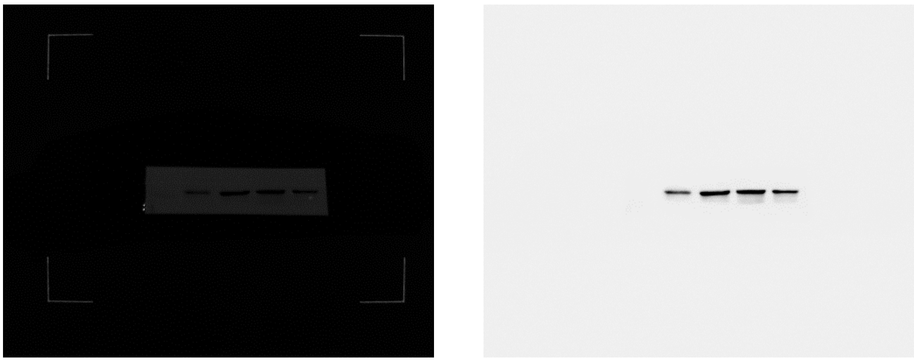

Fig S2 (C) PCNA

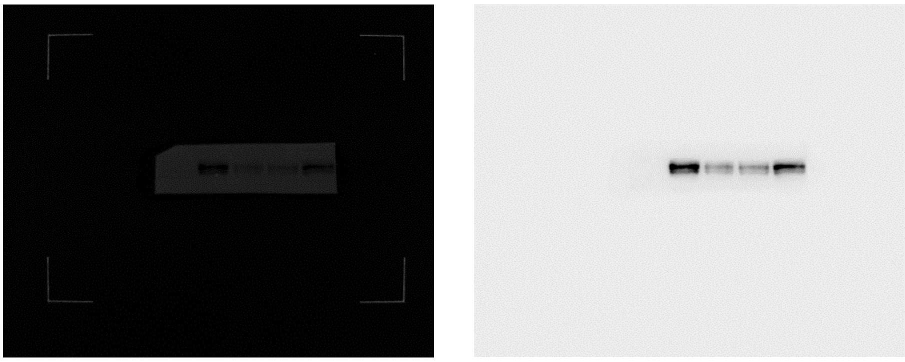

Fig S2 (C) cleaved caspase 3

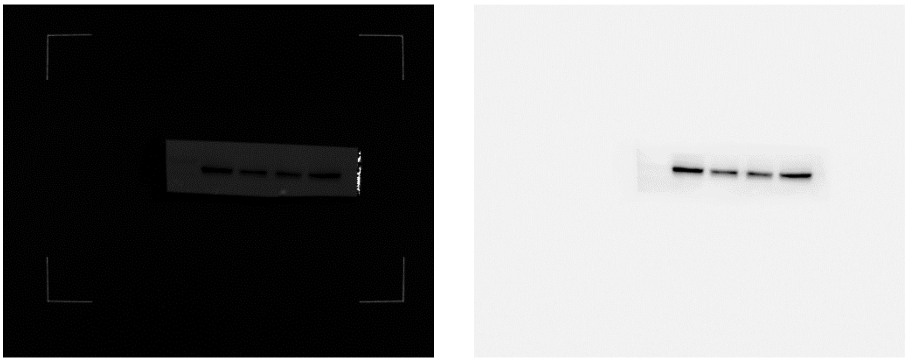

Fig S2(C) Bax

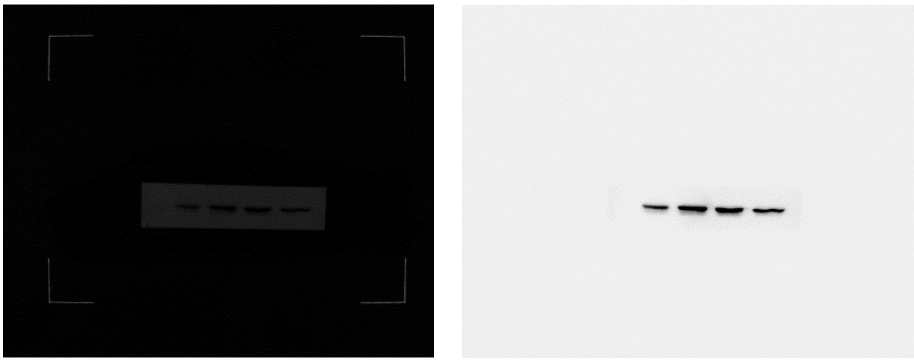

Fig S2(C) Bcl2

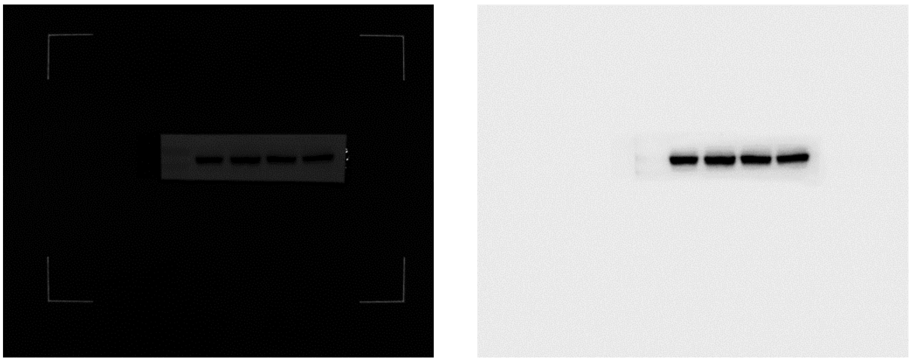

Fig S2(C) β-actin

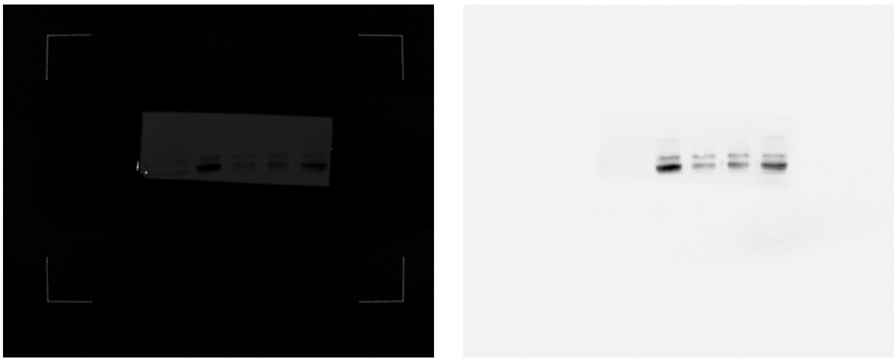

Fig S3(A) LC3B

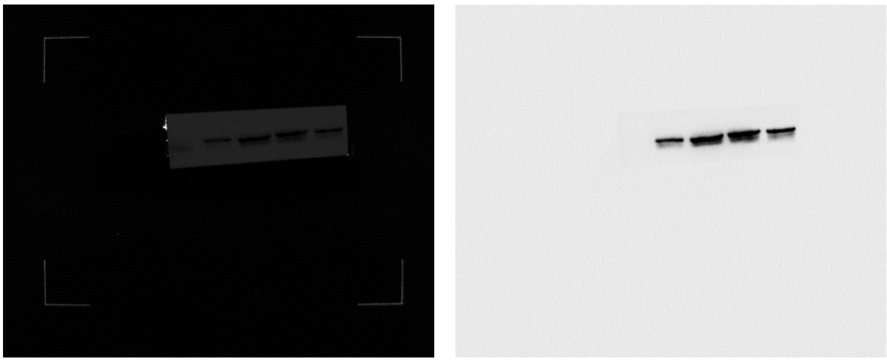

Fig S3(A) p62

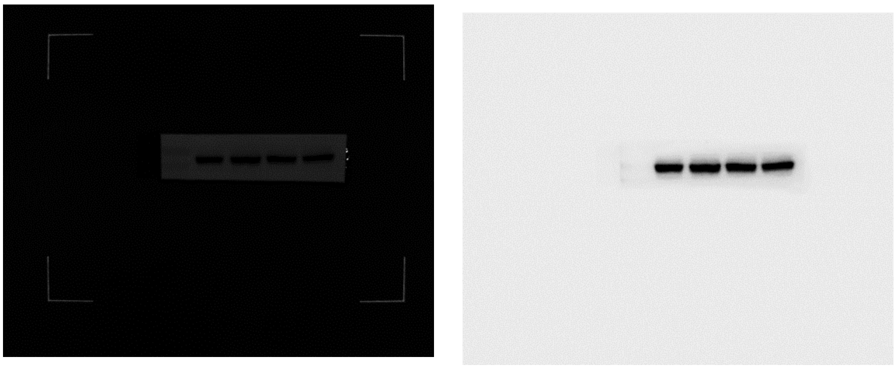

Fig S3(A) β-actin

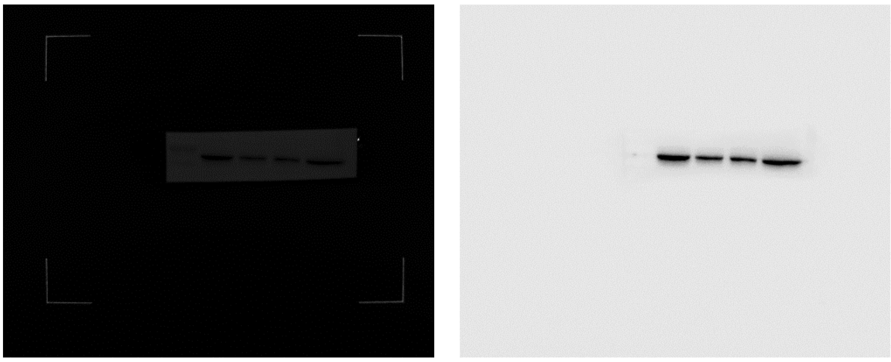
Fig S4(A) BIRC5

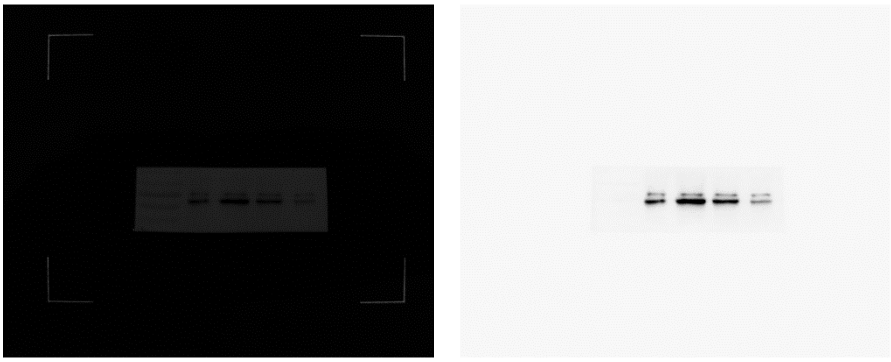
Fig S4(A) LC3B

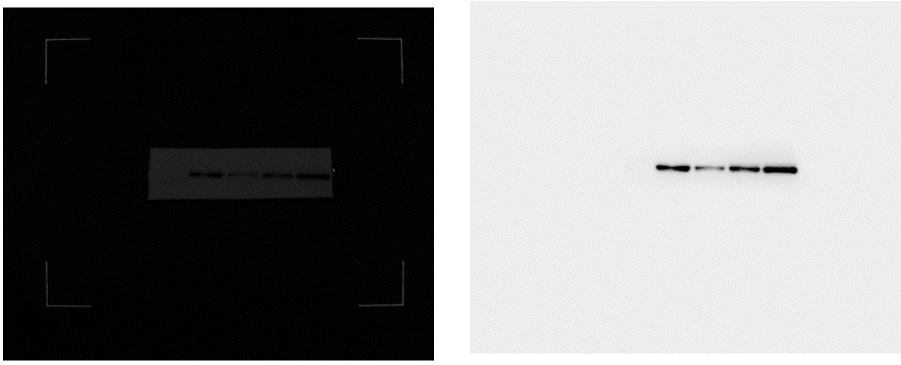
Fig S4(A) p62

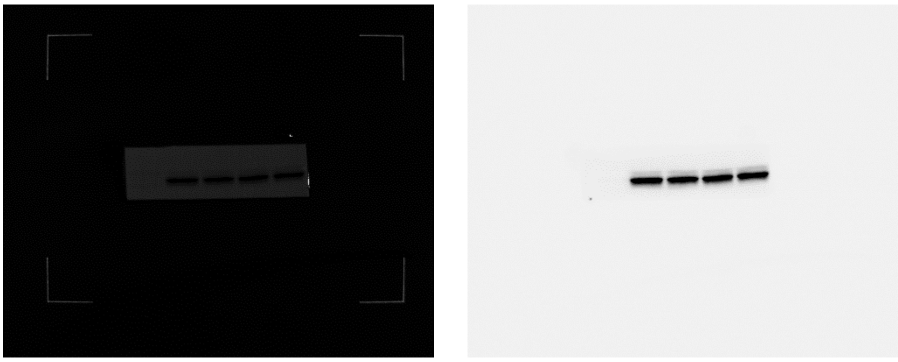
Fig S4(A) β -actin

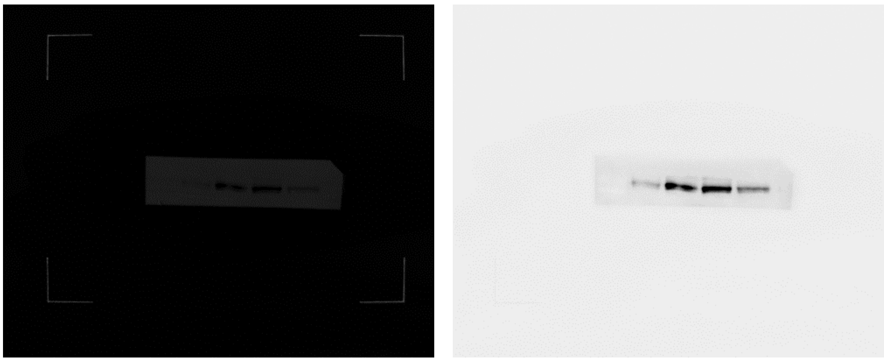

Fig S5(A) p-PI3K

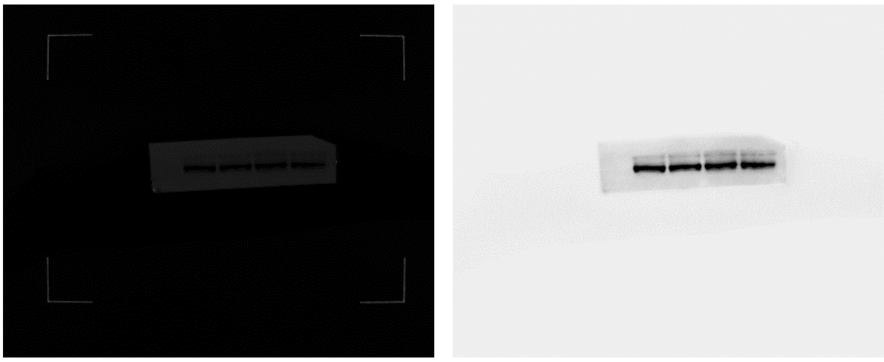

Fig S5(A) PI3K

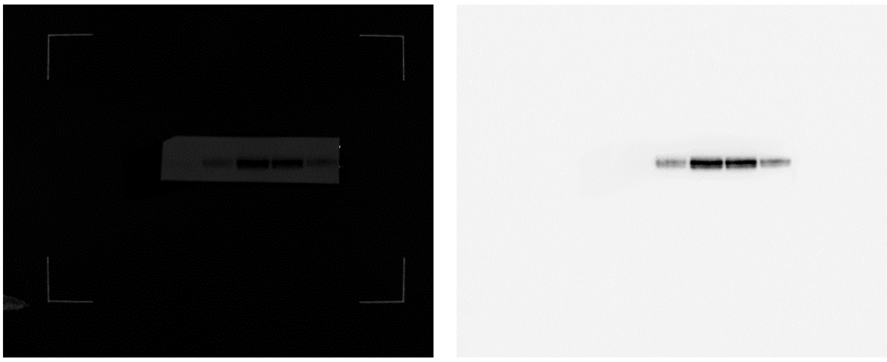

Fig S5(A) p-AKT

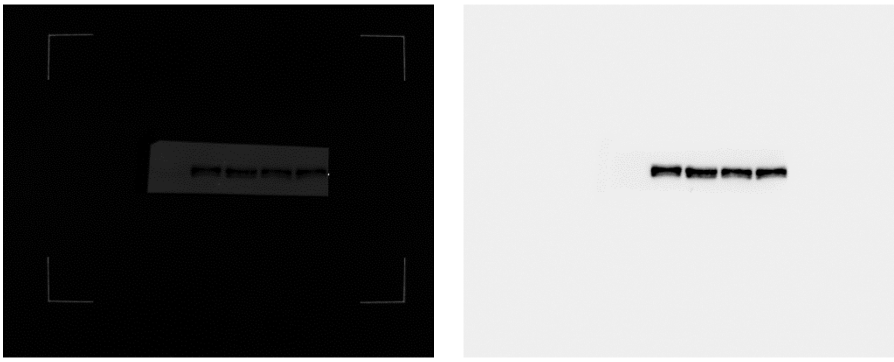

Fig S5(A) AKT

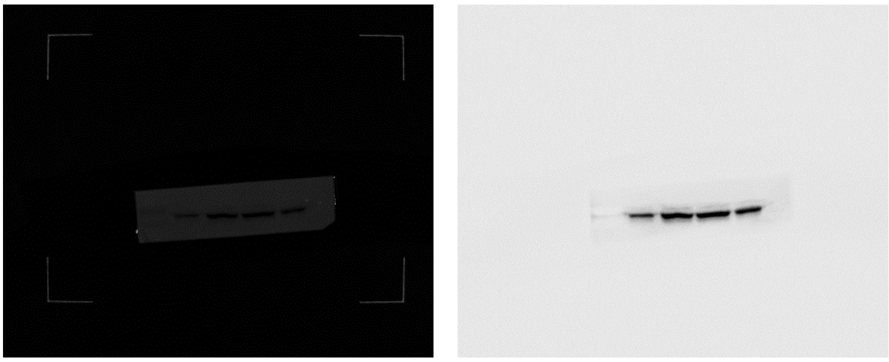

Fig S5(A) p-mTOR

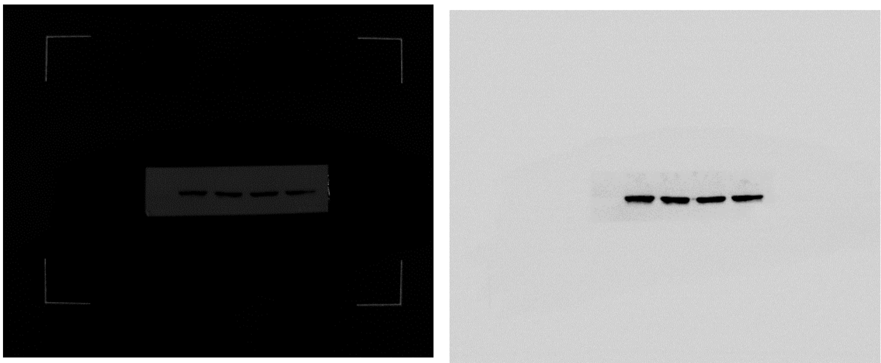

Fig S5(A) mTOR

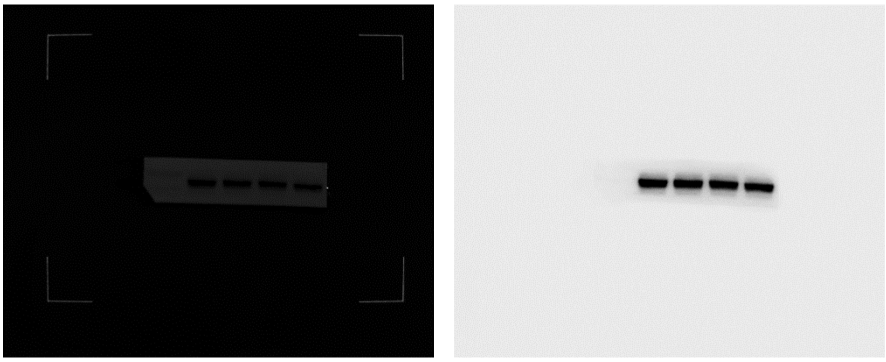

Fig S5(A) β-actin

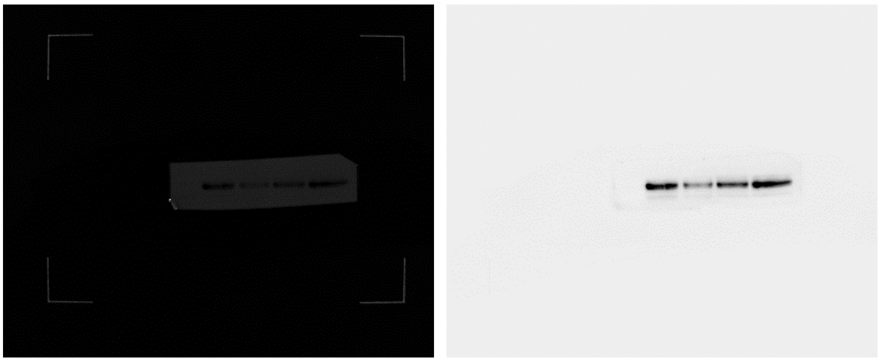

Fig S6(A) p-PI3K

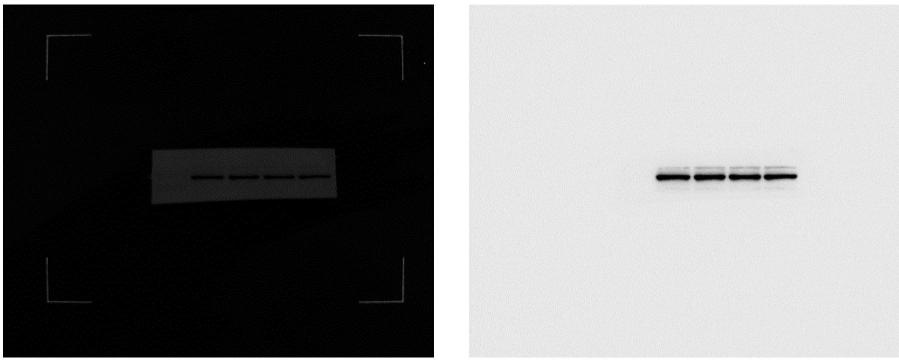

Fig S6(A) PI3K

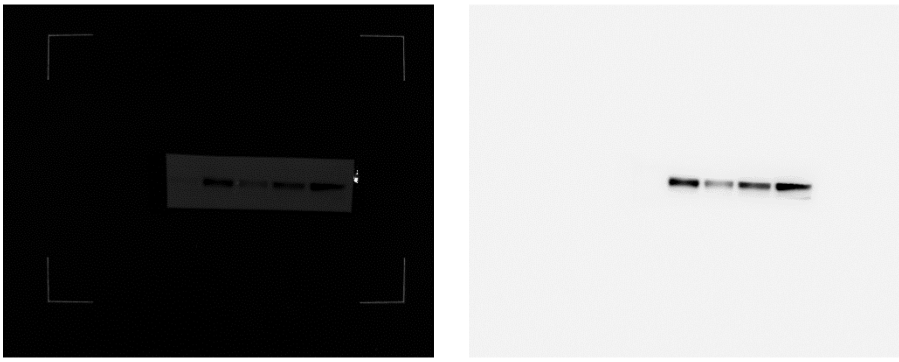

Fig S6（A）p-AKT

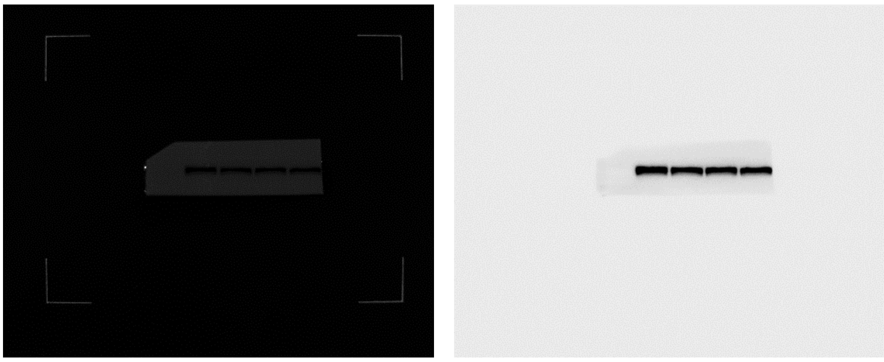

Fig S6 (A) AKT

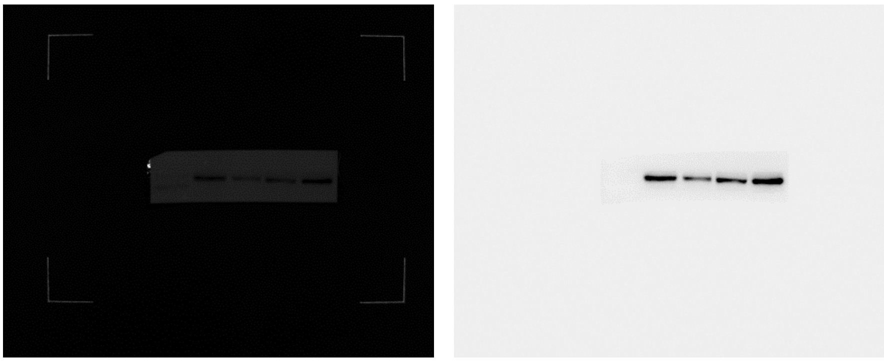

Fig S6(A) p-mTOR

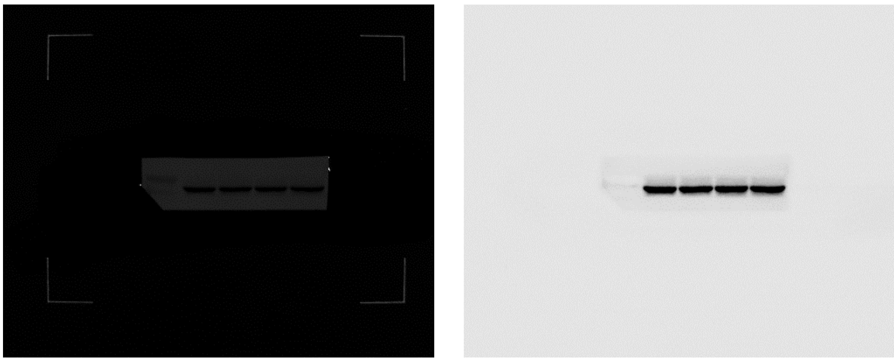

Fig S6(A) β-actin

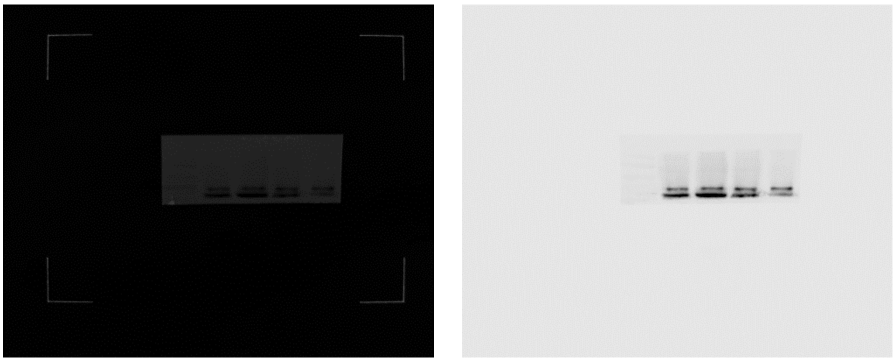

Fig S6（B）LC3BI/LC3BII

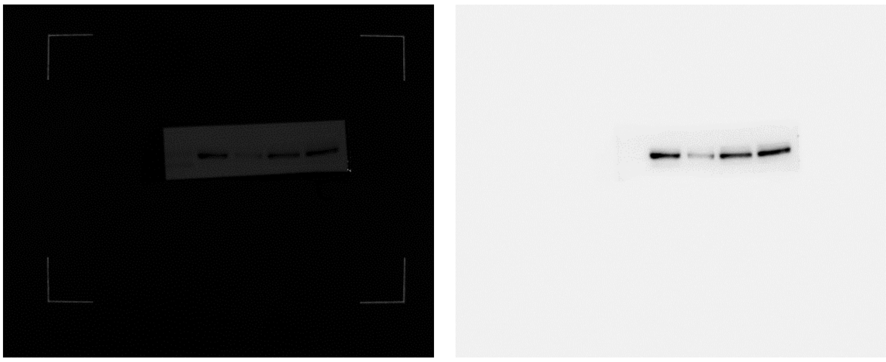

Fig S6（B）p62

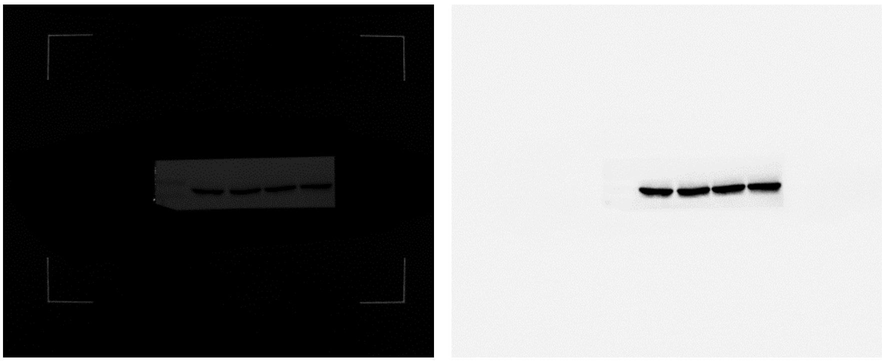

Fig S6（B）β-actin

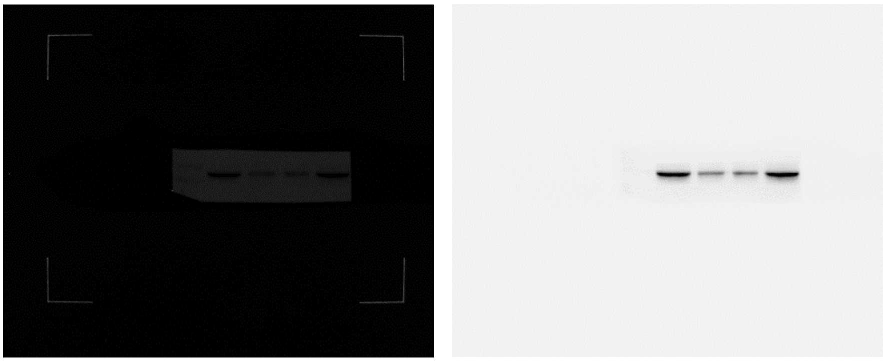

Fig S6（C）BIRC5

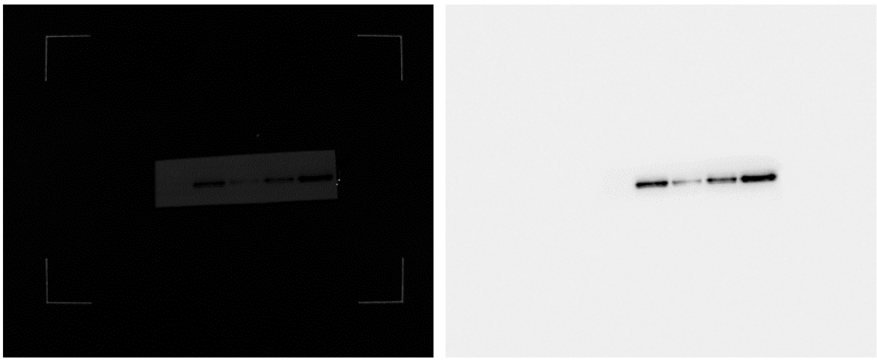

Fig S6（C）PCNA

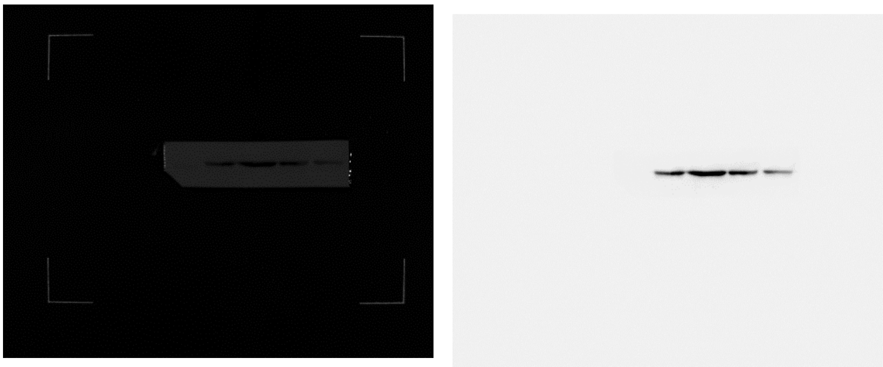

Fig S6（C）cleaved caspase3

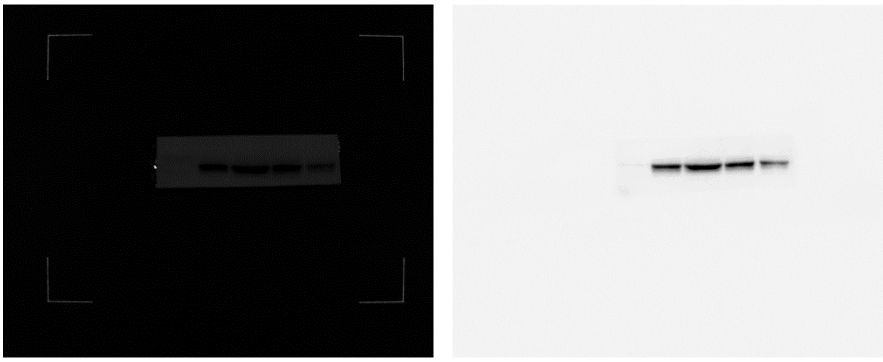

Fig S6（C）Bax

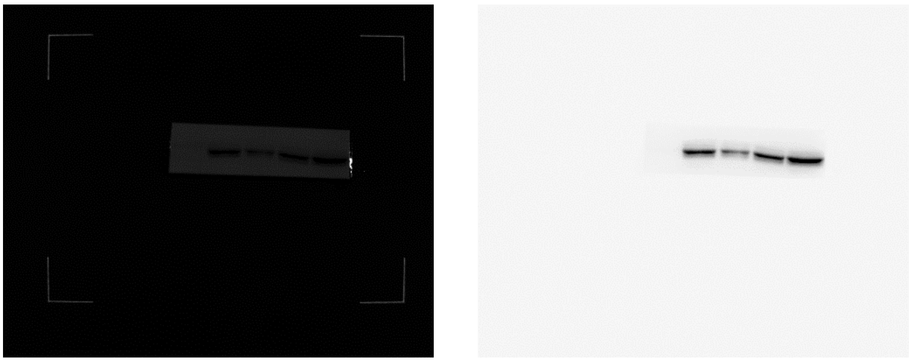

Fig S6（C）Bcl2

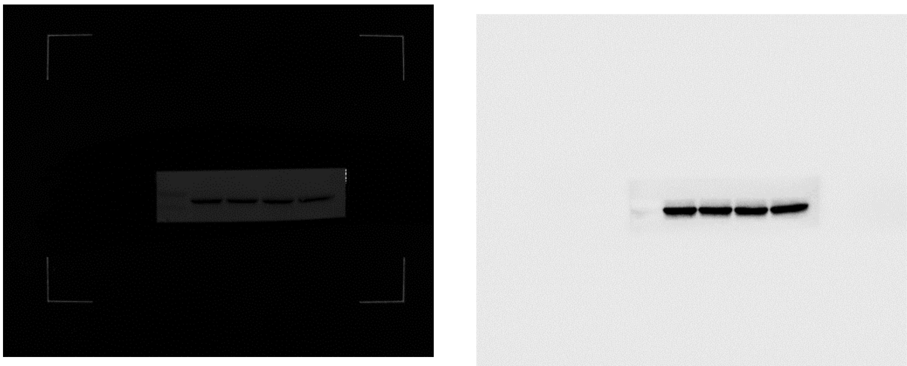

Fig S6（C）β-actin
